# Synthesis and characterization of ternary chitosan–TiO_2_–ZnO over graphene for photocatalytic degradation of tetracycline from pharmaceutical wastewater

**DOI:** 10.1038/s41598-021-03492-5

**Published:** 2021-12-17

**Authors:** Hossein Asadzadeh Patehkhor, Moslem Fattahi, Mohammadreza Khosravi-Nikou

**Affiliations:** 1 Chemical Engineering Department, Abadan Faculty of Petroleum Engineering, Petroleum University of Technology, Abadan, Iran; 2 Department of Gas Engineering, Ahvaz Faculty of Petroleum, Petroleum University of Technology, Ahvaz, Iran

**Keywords:** Chemistry, Engineering, Nanoscience and technology

## Abstract

Various nanocomposites of TiO_2_–ZnO, TiO_2_–ZnO/CS, and TiO_2_–ZnO/CS–Gr with different molar ratios were synthesized by sol–gel and ultrasound-assisted methods and utilized under UV irradiation to enhance the photocatalytic degradation of tetracycline. Characterization of prepared materials were carried out by XRD, FT-IR, FE-SEM, EDX and BET techniques. The TiO_2_–ZnO with the 1:1 molar ratio supported with 1:2 weight ratio CS–Gr (T1‒Z1/CS1‒Gr2 sample) appeared as the most effective material at the optimized operational conditions including the tetracycline concentration of 20 mg/L, pH = 4, catalyst dosage of 0.5 g/L, and 3 h of irradiation time. As expected, the graphene had a significant effect in improving degradation results. The detailed performances of the T1‒Z1/CS1‒Gr2 were compared with ternary nanocomposites from EDX and BET results as well as from the degradation viewpoint. This novel photocatalyst can be effective in actual pharmaceutical wastewater treatment considering the applied operational parameters.

## Introduction

Environmental pollutions and having access to clean water are serious problems worldwide^1,2^. The significant requirement of water in different industries such as chemical, petrochemical, pharmaceutical, textile, paper, and printing industries lead to environmental destruction and threat of ecosystems because of discharge of highly toxic and non-biodegradable wastewater^3^. Moreover, excessive use of pharmaceuticals in recent years and subsequent discharge of pharmaceutical residues and effluents and harmful organic contaminants into the aquatic environment are important issues that need to be addressed seriously^1,4^.

Antibiotics are the widely used as pharmaceuticals and personal care products (PPCP) that can easily transport to the aqueous environments through domestic wastewater, industrial wastewater, runoff, and landfill leachate or directly release veterinary antibiotics via the application in aquaculture. Furthermore, incompletely adsorption of antibiotics by organisms in veterinary, human medicines and discharges 60–90% of them through urine and feces, leading to the abovementioned problem^5–7^. In addition, due to the increasing use of antibiotics in veterinary and human medicine, antibiotic-resistance bacteria develop at low concentrations of antibiotics in the environment in which lead to the ineffectiveness of them in the treatment of several diseases. It is noteworthy that most antibiotics are highly polar and non-volatile that they persist in environmental matrices. However, antibiotics are used as antibacterial, antiviral, antifungal, and anti-tumor agents and can be classified according to spectrum, mechanism of action, or chemical structure^1,5,8^. Therefore, the efficient removal of different contaminants such as pharmaceuticals is considered a global challenge^7^.

Various chemical and physical methods such as adsorption, ultra-filtration membrane, reverse osmosis, electrocoagulation, ion exchange, and biological treatment have been extensively utilized in recent years to remove organic compounds and pharmaceuticals from wastewater^3,5,8^. The need to develop feasible, environmental-friendly, sustainable, and cost-effective technologies is felt more than ever due to the inability of conventional sewage treatment plants to completely remove pharmaceutical contaminants^1,2^. Advanced oxidation processes (AOPs) are alternative and more efficient techniques that generate the powerful oxidant of active hydroxyl free radicals (•OH) that leads to breaking down the coarse contaminant molecules and efficient mineralization of organic compounds^3,9^.

Tetracycline (TC) antibiotics are one of the largest groups of widely used antibiotics worldwide in terms of production and consumption that were discovered in the 1940s^4,9^. The complex structure of TC, hydrophilicity of large molecules, solubility in the ranges of g/L, emission to the environment caused by poor absorption by humans and animals, and excretion of large amounts are the distinctive properties necessitate for efficient removal of it from wastewater that is practical through the photocatalytic processes^7,10,11^.

Heterogeneous photocatalysis by employing semiconductor particles is the most attractive and efficient choice that receives much attention in air purification and decomposition of organic pollutants such as TC in an aqueous environment^3,12,13^. Catalyst concentration, wavelength, radiation intensity, pH, and water matrix are the main parameters that affect the performance of this process^5^. Despite all the outstanding features and merits of semiconductor metal oxides as ubiquitous heterogeneous photocatalysts, there are some inherent drawbacks when using them alone. Therefore, many attempts such as their combinations with other components have been made to overcome these drawbacks^14,15^.

Titanium dioxide (TiO_2_) has attracted much attention as excellent semiconductor metal oxide due to its significant properties and high potential in photocatalytic applications. Anatase, rutile, and brookite are three phases of TiO_2_ crystals where the anatase phase is most favorable and practical because of its phenomenal photocatalytic activity^6,12,16^. To improve the photocatalytic activity of TiO_2_ and to overcome some of its drawbacks such as high electron–hole recombination rate and large bandgap, multi-semiconductor hybrid materials could be applied as a modification approach^15,17,18^. Zinc oxide (ZnO) is another promising semiconductor that couples with TiO_2_ to compensate its afore-mentioned limitations. Moreover, ZnO nanoparticles absorb more light photons than TiO_2_ nanoparticles under the same ambient conditions. The photocatalyst composite of TiO_2_–ZnO is known as a high-performance photocatalyst because of the similarity of these semiconductor nanostructures in photodegradation mechanism and the efficient accomplishment of electron–hole pair separation^19–21^. Although numerous researches on the removal of different organic pollutants from water using TiO_2_–ZnO as photocatalyst nanocomposite has already been done, the interfacial charge transfer rate of ZnO is low and both of mentioned photocatalysts have a large bandgap and high electron–hole recombination rate. Furthermore, both of them have a great tendency to aggregation, especially at high concentrations. Hence, to enhance the photocatalytic activity and to solve the aggregation problem, the addition of different compounds such as various biopolymers and carbon materials to the semiconductors as solid supports is a suitable approach^7,15,22^.

Separation and recycling of the used photocatalysts are considerable problems in practical using. Solid supports of the catalysts play a significant role in facilitating these through catalyst immobilization^7^. It was also reported that support materials increase the surface area of catalytic systems and cause improvement in hydrophobicity, thermal, hydrolytic, and chemical stability of the catalysts^23^. Chitosan (CS), an attractive and functional polymeric support, is a natural biopolymer obtained by deacetylation of polysaccharide chitin derived from structural components of marine organisms such as crabs and shrimps^24,25^. CS’s structure contains highly reactive hydroxyl groups and amino groups that lead to unique characteristics^3,24^. Moreover, CS/metal oxides photocatalytic system has some advantages such as enhancement of the area of light exposure, improvement of the synergistic photocatalysis, long-term reusability, elimination of the need to filter the treated water, and sustainable usage. However, this system has some limitations considering the protonation of amino groups of the CS when using as the photocatalyst where protonation leading to decreased adsorption of the pollutants because the unprotonated amino group of the CS plays the main role in adsorbing pollutants. When utilizing this photocatalytic system, loss of catalysts and secondary pollution could also happen due to the difficulty of recovery the evenly dispersed catalysts in water. Several methods to modify the CS and overcome the limitations of CS/metal oxides photocatalyst have been suggested^23^.

In recent years, numerous studies about improved photocatalytic performance by introducing carbon materials have already been reported. The carbon-supported photocatalytic complex exhibits excellent optical response properties and makes reengineering the bandgap possible^23^. Graphene (Gr), as outstanding two-dimensional support for nano-metal oxides catalyst, has attracted much attention due to its large surface areas (theoretically about 2600 m^2^/g). Also, Gr could be effective in minimization of the recombination issues^15,26^.

In the current study, the novel photocatalyst nanocomposites including the TiO_2_–ZnO supported on CS–Gr were synthesized and compared with only CS support and non-supported TiO_2_–ZnO. The main purpose of this research was to enhance the photocatalytic degradation of TC via the synthesized nanocomposites. As far as the authors know, although numerous efforts to remove tetracycline from water using TiO_2_ and ZnO and their composites have been made, reports on utilizing of the two support materials simultaneously are scarce. The influence of various operational parameters was examined to achieve the optimum conditions and enhanced photocatalytic degradation. Moreover, the characteristics of the as-prepared nanocomposites were evaluated via different characterization techniques.

## Materials and methods

### Materials

HPLC-grade tetracycline powder (C_22_H_24_N_2_O_8_.xH_2_O, ≥ 98% purity, MW: 444.43), and chitosan (high molecular weight) were purchased from Sigma-Aldrich, USA. Tetra-butyl titanate (C_16_H_36_O_4_Ti, ≥ 98%, for synthesis, TBT), ethanol (C_2_H_6_O, absolute grade), nitric acid (HNO_3_, 65%), hydrochloric acid (HCl, fuming 37%), sodium hydroxide (NaOH), and acetic acid (glacial 100%) were obtained from Merck, Germany, and purified sodium chloride (NaCl) was purchased from an Iranian company. Zinc acetate dihydrate (Zn(CH_3_COO)_2_.2H_2_O, ≥ 98%, ZnAc) and graphene nanoplatelet (+ 99.5%, 2–18 nm with 32 layers) were purchased from Sigma-Aldrich, USA and US Research Nanomaterials, respectively.

### Preparation of TiO_2_-ZnO nanocomposites

#### Synthesis of TiO_2_ nanoparticles via sol–gel method

Initially, the precursor solution was prepared by adding dropwise 5 mL of TBT in 20 mL of absolute ethanol under vigorous magnetic stirring for 30 min (solution A). A mixture of 5 mL of absolute ethanol, 3 mL of distilled water, and 0.5 mL of nitric acid (solution B) was then added dropwise into solution A under constant stirring, which resulted in a pH value of about 2. Stirring of achieved transparent yellow sol was continued for 5 h at room temperature and aged for 8 h to form a white and uniform gel. This obtained gel was dried at 80 °C in an oven and converted to TiO_2_ nanocrystalline. After crushing the crystals to a fine powder, the obtained TiO_2_ nano-powder was calcined at 400 °C for 2 h in a muffle furnace and used to prepare nanocomposites. This synthesis procedure obtained from different references^27–29^ to provide the TiO_2_.

#### Synthesis of TiO_2_–ZnO nanocomposites

The synthesis process of TiO_2_–ZnO nanocomposites was followed by modifying the Prasannalakshmi et al. procedure^30^ and via the ultrasound-assisted sol–gel method. In this study, the preparation of a homogeneous solution of ZnO was carried out as follows: 2060 mg of ZnAc was added into 40 mL of double-distilled water and completely dissolved in it by magnetic stirring. Next, 40 mL of prepared 0.5 M of NaOH solution was added dropwise in the above solution under magnetic stirring at a higher speed. Next, this ZnO solution and 750 mg of already prepared TiO_2_ nanoparticles were introduced together under sonication for 30 min. The whole mixture was stirred continuously for 4 h to get a gel. The obtained gel was washed several times and then dried at 90 °C. Finally, this TiO_2_–ZnO sample with a molar ratio of 1:1 was annealed at 550 °C for 4 h and was named T1–Z1. To synthesize TiO_2_–ZnO with a molar ratio of 2:1, the same procedure was performed exactly with the difference in the TiO_2_ quantity that was equaled 1500 mg and was named T2–Z1. These nanocomposites were synthesized several times for later use.

### Preparation of TiO_2_–ZnO/CS nanocomposites

#### Chitosan solution preparation

The CS solution prepared with a concentration of 5 mg/mL (0.5% w/v) according to Bhanvase et al. report^24^. First, a mixed solution of acetic acid (80 mL, 0.1 M) and NaCl (20 mL, 0.2 M) was prepared, and afterward, 500 mg of CS was dissolved in it overnight that resulted in the formation of CS suspension. The obtained suspension was sonicated for 1 h to get a viscous solution of fully dissolved CS.

#### Synthesis of TiO_2_–ZnO/CS nanocomposites

The TiO_2_–ZnO/CS ternary nanocomposites were synthesized according to the Bhanvase et al.^24^ via an ultrasound-assisted preparation method. For this purpose, as-prepared T1–Z1 and T2–Z1 composites were used, and also, CS quantity was considered 5 wt.% of the ternary composite. To synthesize the T1–Z1/CS nanocomposite, 10 mL of CS solution was taken as support for loading the T–Z. The loading of T–Z was accomplished by adding 950 mg of it into the CS solution under sonication. The 20 mL of NaOH of 1M was added dropwise under sonication immediately, which resulted in the complete loading of T–Z. The resultant colloidal solution was sonicated at room temperature for 1 h. In the final step, the obtained sample was filtered and washed with distilled water several times until pH reached neutral value, and then drying was carried out. This dried sample was crushed to get a fine powder. The process was performed exactly as before in association with the T2–Z1/CS nanocomposite.

### Preparation of TiO_2_–ZnO/CS–Gr nanocomposites

Four types of the novel photocatalyst nanocomposites of T–Z/CS–Gr with various ratios were synthesized via the self-assembly of components of these nanostructures. So, the already prepared T1–Z1 and T2–Z1 were combined with CS–Gr as support, with the CS–Gr weight ratio of 1:1 and 1:2, where CS was 5 wt.% of the whole composite like before, and Gr was 5 wt.% and 10 wt.%, respectively. For preparation of T1–Z1/CS1–Gr1 nanocomposite, first, 50 mg of Gr was dispersed via sonication in 10 mL of CS solution (5 mg/mL) for 45 min. After obtaining the well-dispersed CS–GR colloid, 900 mg of T1–Z1 composite was loaded slowly on the above colloidal solution, and 10 mL of 1M of NaOH was added dropwise immediately. In addition, both previous processes were under sonication. Next, this mixture was sonicated at room temperature for 75 min, and finally, this gray sample was filtered and washed with distilled water several times to decrease the pH to its neutral value. The obtained product was crushed to a fine powder after drying at 70 °C. The T2–Z1/CS1–Gr1 nanocomposite was prepared according to the same previous procedure. Furthermore, to prepare the T1–Z1/CS1–Gr2 and T2–Z1/CS1–Gr2 nanocomposites the amounts of Gr was 100 mg, and the T–Z composites was 850 mg. Other conditions and parameters were the same.

### Characterizations

The structure of some prepared samples was studied using the powder X-ray diffraction (XRD) by a Philips PW 1730 (Netherlands) diffractometer with Cu ${\mathrm{K}}_{\mathrm{\alpha }}$ (λ = 1.54056 Å) radiation. The diffraction angle (2θ) range in this analysis was 10°–90°. Fourier-transform infrared spectroscopy (FT-IR) of some samples was carried out on a Thermo AVATAR FTIR spectrometer (USA) in the wavenumber range of 400–4000 cm^-1^. The structural morphology and the elemental composition of nanostructures were assessed by the FESEM MIRA III instrument (TESCAN, Czech Republic) with the techniques of field emission scanning electron microscopy (FE-SEM) and energy-dispersive X-ray (EDX) spectroscopy, respectively. The specific surface area and the porosity of most active photocatalyst was compared with similar ternary nanocomposite by N_2_ adsorption at 77 °K on a BEL PREP VAC II high precision surface area and pore size analyzer (BELSORP-MINI II model, BEL, Japan) and estimated using the Brunauer–Emmett–Teller (BET), and the Barrett–Joyner–Halenda (BJH) methods, respectively.

### Photocatalytic activity

The photocatalytic performance of as-prepared nanocomposites was investigated by degrading tetracycline at room temperature and default experimental conditions. A closed chamber illuminated with five UV lamps (365 nm) equipped with a magnetic stirrer was used that the photocatalytic reactions were performed in a batch reactor. TC powder has been completely dissolved in distilled water to prepare the considered concentrations. In all cases, 100 mL of already prepared solution of TC pollutant was used, and the pH value was adjusted by HCl and NaOH diluted solutions; also, the suspension containing TC solution and catalyst was stirred in the dark for 1 h before using UV light to reach the adsorption-desorption equilibrium. In the final step of photocatalytic tests, to estimate the removal percentage of TC caused by reaction with the catalyst under UV, the used catalyst had been completely separated by centrifuging (UNIVERSAL 320 centrifuge, PIT co. Iran) and filtering; then, the required amount of initial and degraded TC solutions had been transferred to a UV-vis spectrophotometer (UNICO SQ-2800). Consequently, the absorbance of solutions in the wavelength of 358 nm was measured and compared together, and eventually, the percentage of removal efficiency was calculated by the following equation:

1 $${\text{Removal }}\left( {\text{\% }} \right) = \frac{{{\text{C}}_{0} - {\text{C}}_{{\text{d}}} }}{{{\text{C}}_{0} }} \times 100$$

where ${\text{C}}_{0}$ and ${\text{C}}_{{\text{d}}}$ are the initial and degraded TC concentrations, respectively.

### Experimental procedure design

As a first step in the degradation assessment, photocatalytic tests were carried out with 30 min intervals by the binary and ternary nanocomposites. Then, the experiments were performed at the obtained equilibrium time for specifying the best nanocomposite in terms of ratios between components. In both the abovementioned stages, other parameters were considered as TC solution with 10 mg/L concentration, pH of 7, and 0.5 g/L catalyst dosage. Afterward, pH, initial TC concentration, and catalyst dosage were changed to different levels to reach the optimum conditions and the most efficiency in the removal of TC. Hence, the photocatalytic tests were repeated at pH values of 4, 6, and 8, and other parameters were the same as before. Next, tests were performed at optimum pH, with variations in the initial TC concentrations including the 15, 20, 25, 30 mg/L. Finally, after specifying the optimum parameters of TC solution, catalyst dosage was considered in the values of 0.3 and 0.7 g/L to achieve the highest efficiency in the photocatalytic degradation of TC.

## Results and discussion

### Characterization of catalysts

**Figure 1:**
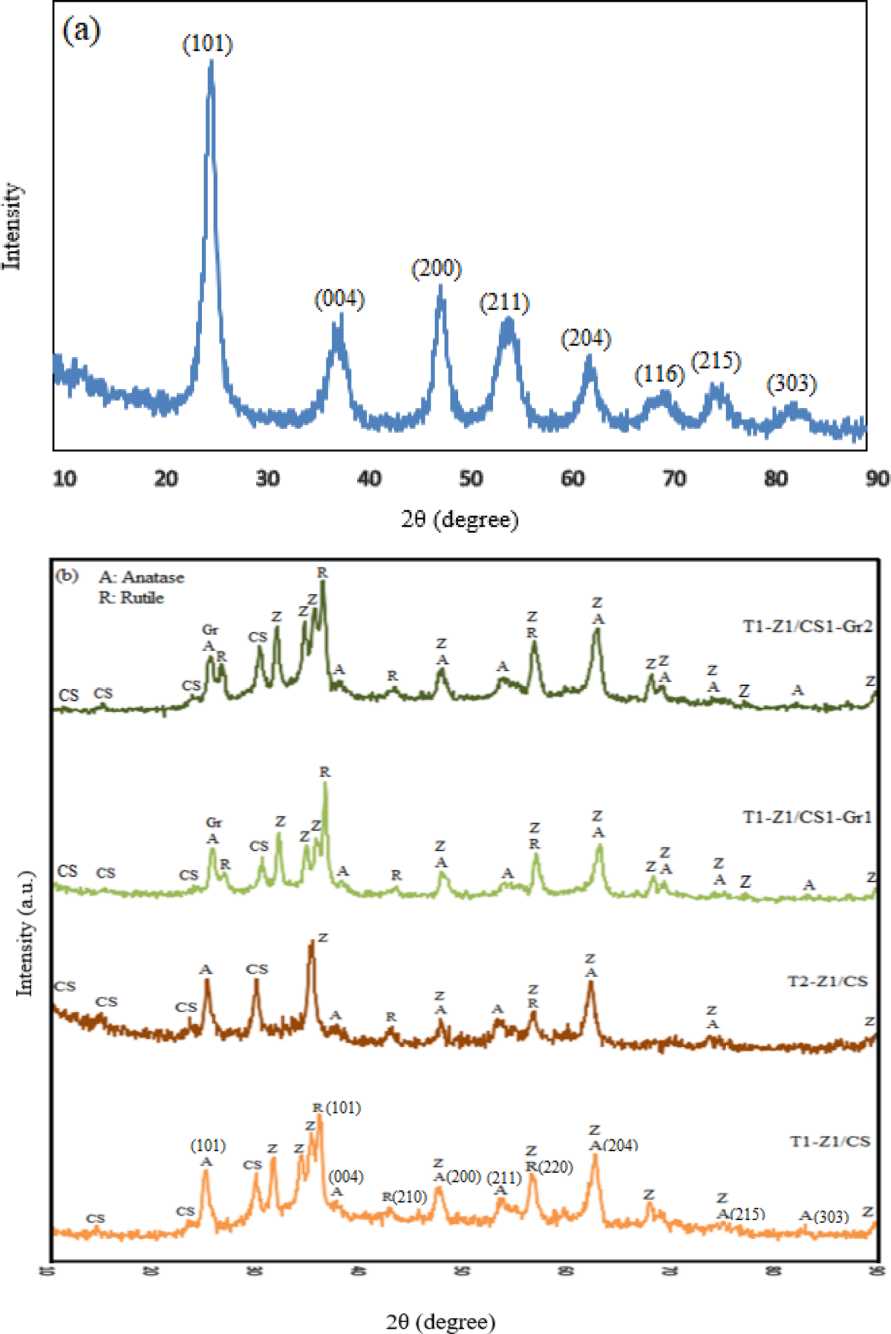
XRD patterns of (**a**) pure TiO_2_ (**b**) composite samples.

The XRD patterns of prepared TiO_2_, T1–Z1/CS, T2–Z1/CS, T1–Z1/CS1–Gr1, and T1–Z1/CS1–Gr2 are shown in Fig. 1a, b. According to these patterns, it could be found that the TiO_2_ has appeared mainly in the anatase crystalline phase. In other words, some phase transformations from anatase to rutile have been occurred due to the heat treatment and change in pH value during its participation in the synthesis process of composites. It is noteworthy that the presence of TiO_2_ in the mix-crystal structure leads to higher photoactivity than its single-phase structure^7^. Achieved sharp diffraction peak located at the 2θ of 25.65° angle position and other existent diffraction peaks at 38.4°, 48.2°, 55°, 62.8°, 70°, 74.8°, and 83° in the XRD pattern of TiO_2_ indicate the standard peaks of the anatase TiO_2_. These peaks are respectively related to the (101), (004), (200), (211), (204), (116), (215), and (303) crystallographic planes according to the JCPDS 21–1272^31–33^. Due to the diffraction peaks of CS supported and CS–Gr supported T–Z composites, it might be concluded that the interaction between the components was performed properly. Some diffraction peaks of anatase ${\mathrm{TiO}}_{2}$ have slightly shifted in the T1–Z1/CS composite, and it seems that some peaks of the anatase and rutile were overlapped with the ZnO diffraction peaks. The peaks at the positions of 36.1°, 42.9°, and 56.5° can be assigned to the (101), (210), and (220) planes of the rutile phase (JCPDS No. 21-1279), respectively^27,34^. Moreover, ZnO exhibits the distinctive peaks located at angle position of 31.75°, 34.5°, 35.35°, and 89.7° attributed to the (100), (002), (101), and (203) hexagonal wurtzite ZnO planes, respectively according to the JCPDS Card No. 36–1451 content^35^; also, it may be overlapped at the specified positions on Fig. 1. The diffraction peaks corresponding to the crystalline phase of CS are present at 10.3°, 14.65°, and 23.45° weakly^3,25,28^; besides, the sharp peak at 30° can be related to CS presence^36^. From the XRD pattern of T2–Z1/CS, it is evident that some diffraction peaks of ZnO disappeared; besides, the change in the molar ratio has led to the changes in the intensities values that can be caused by the variation in the reaction and bonding between components. The previous diffraction peaks are present in the T1–Z1/CS1–Gr1 and T1–Z1/CS1–Gr2 composites, with a slight change in the intensity. Additionally, the new peaks appeared at ~ 27°, and 77° can be assigned to the rutile phase of TiO_2_ and (202) named plane of wurtzite ZnO, respectively^34,35^; meanwhile, the diffraction peak present at ~ 25.5° can be attributed to the (002) plane of Gr^13^.

**Figure 2:**
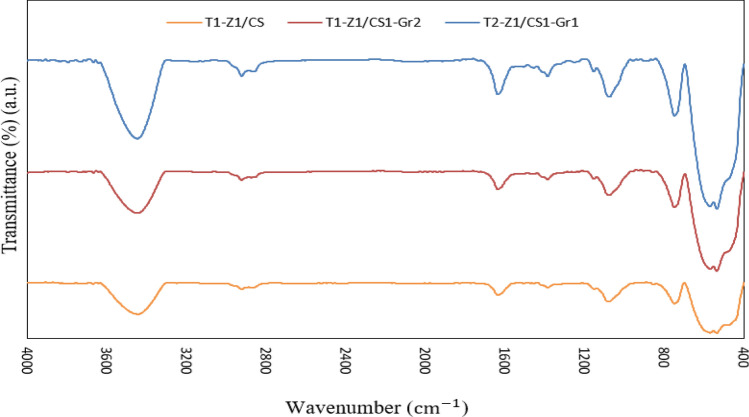
FTIR spectra for nanocomposites in this study.

The FTIR spectra of T1–Z1/CS, T1–Z1/CS1–Gr2, and T2–Z1/CS1–GR1 composites have been obtained according to Fig. 2 to assess the functional groups. In the spectrum of ternary composite, CS exhibits clear characteristic absorption peaks due to its original functional groups. These peaks are located at wavenumbers of ~ 3445 cm^-1^, ~ 1636 cm^-1^, and ~ 1078 cm^-1^ and respectively are attributed to the overlapping of the –NH and –OH stretching vibrations^32,37,38^, C = O stretch of –NHCO– in amide group (amide I)^31,37,39^, and vibrational motions of –C–O–C in glucose unit^3,38^. Also, two other bands with a weak intensity have appeared at ~ 2924 cm^-1^ and ~ 1384 cm^-1^, which are attributed to the aliphatic C–H stretching vibration and asymmetric C–H stretch bending of –CH_3_ group, respectively^3,37,38^. The achieved peaks in the fingerprint region confirm the presence of TiO_2_ and ZnO and exhibit their successful incorporation into the polymer structure; therefore, the peaks located at ~ 749 cm^-1^, ~ 569 cm^-1^ and ~ 536 cm^-1^, and ~ 484 cm^-1^ could be respectively assigned to Zn–O–Ti, Ti–O, and Zn–O stretching vibrations^21,34,35^. In the spectrum of T1–Z1/CS1–Gr2 composite, the previous bands between 4000 and 1000 cm^−1^ have been broader and slightly shifted that are good signs related to the existence of Gr. Moreover, the peaks located at ~ 3447 cm^-1^, ~ 1635 cm^−1^, and ~ 1069 cm^-1^ could be attributed to the vibrational motions of OH moieties, NH_2_ group, and C–O–C related to the presence of Gr^39^. However, the band located at the below 500 cm^-1^ is not to be observed in this sample which can be caused by the change in the interactions between components. Compared with previous composites, two new peaks have formed at ~ 1155 cm^−1^ and ~ 884 cm^−1^ in the spectrum of T2–Z1/CS1–GR1 composite; these peaks are related to the saccharides structure of the CS and Zn–O–Ti stretching motion in the T–Z nanocomposite, respectively^24,31^. Furthermore, no considerable effect is observed on the intensity of the bands with the change in the molar ratio of the T–Z composite, which is in agreement with the literature work^34^.

**Figure 3:**
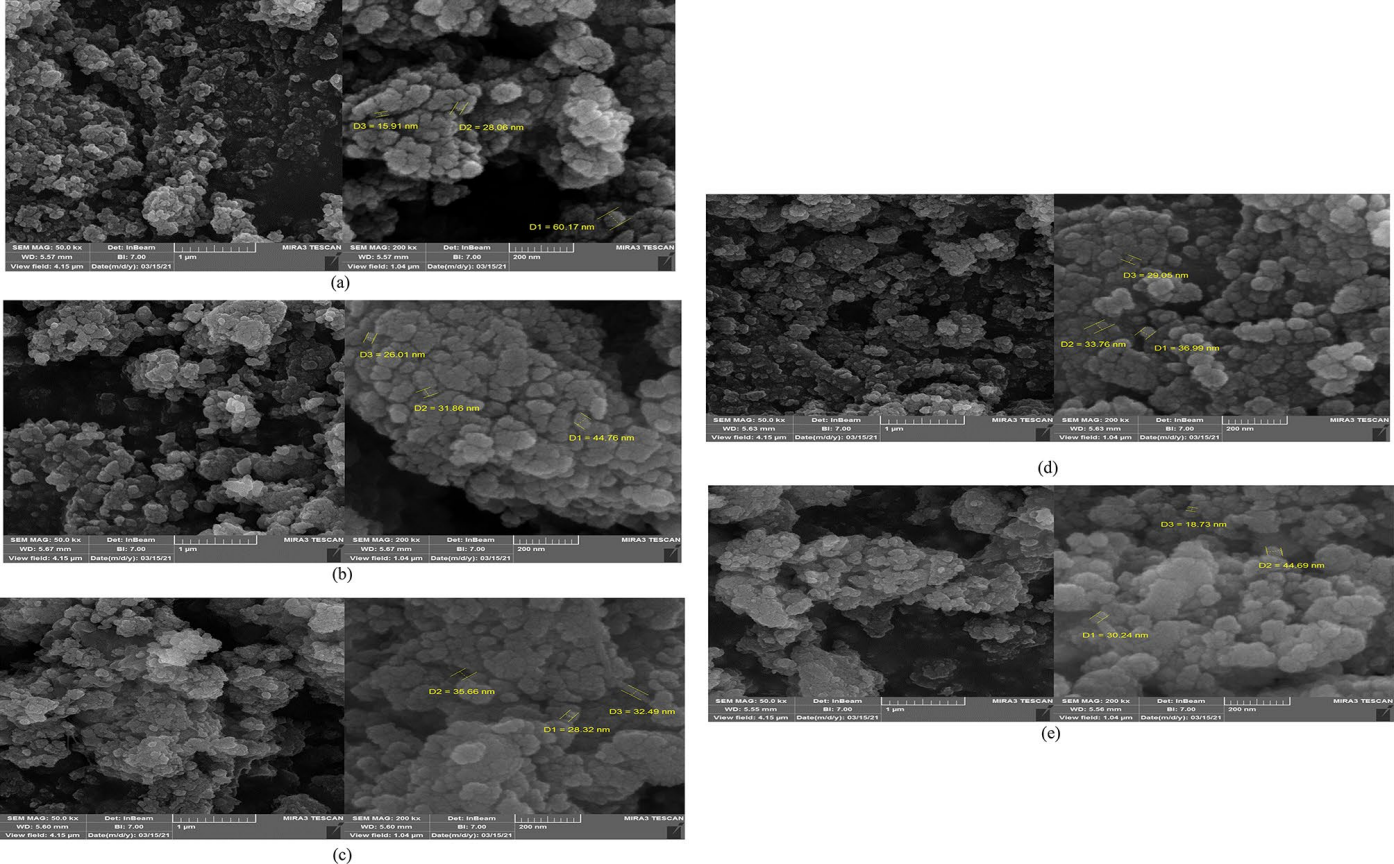
FE-SEM images of (**a**) TiO_2_ nanoparticle (**b**) T1‒Z1 (**c**) T1‒Z1/CS (**d**) T2‒Z1/CS (**e**) T1‒Z1/CS1‒Gr2 nanocomposites.

One of the main influencing parameters in utilizing catalysts is the surface morphology. Hence, the morphology of as-prepared TiO_2_ nanoparticles and some multiple prepared nanocomposites were investigated, and their particle size was marked on the images. Figure 3a shows the obtained spherical TiO_2_ nanoparticles. In the FE-SEM images of the T1–Z1 binary composite, according to Fig. 3b, TiO_2_ and ZnO nanoparticles are seen almost uniformly. According to the results of literature work^34^, the morphology of T–Z systems to be significantly affected by the molar ratio of reagents. So that, in the systems with higher TiO_2_ content, spherical particles appear that have a great tendency to agglomerate. With an equal molar ratio in this composite, although a relative tendency to agglomerate exists, ZnO particles are easily recognizable. Due to the obtained images for T1–Z1/CS ternary composite in Fig. 3c, the presence of TiO_2_ and ZnO particles on the CS is seen; and seemingly ZnO particles are rod-shaped, which can be caused by the repeated synthesis of the T–Z composite. The effect of high TiO_2_ content on the T2–Z1/CS composite is visible clearly according to Fig. 3d. Finally, the obtained images for T1–Z1/CS1–Gr2 composite have been shown in Fig. 3e. The interaction between these components occurred due to the negative charges of graphene sheet caused due to the existence of COOH, C=O, and OH functional groups, being cationic of CS polymer, and the positive charge of metal ions^40^.

**Figure 4:**
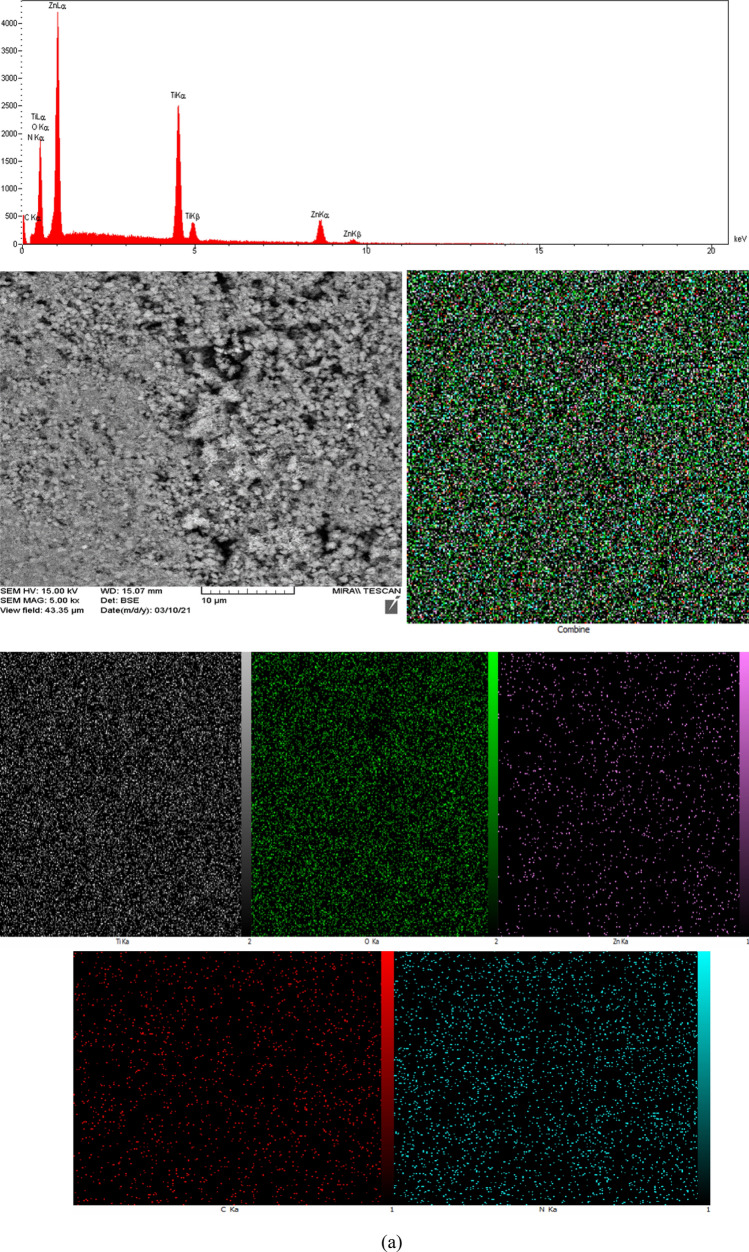

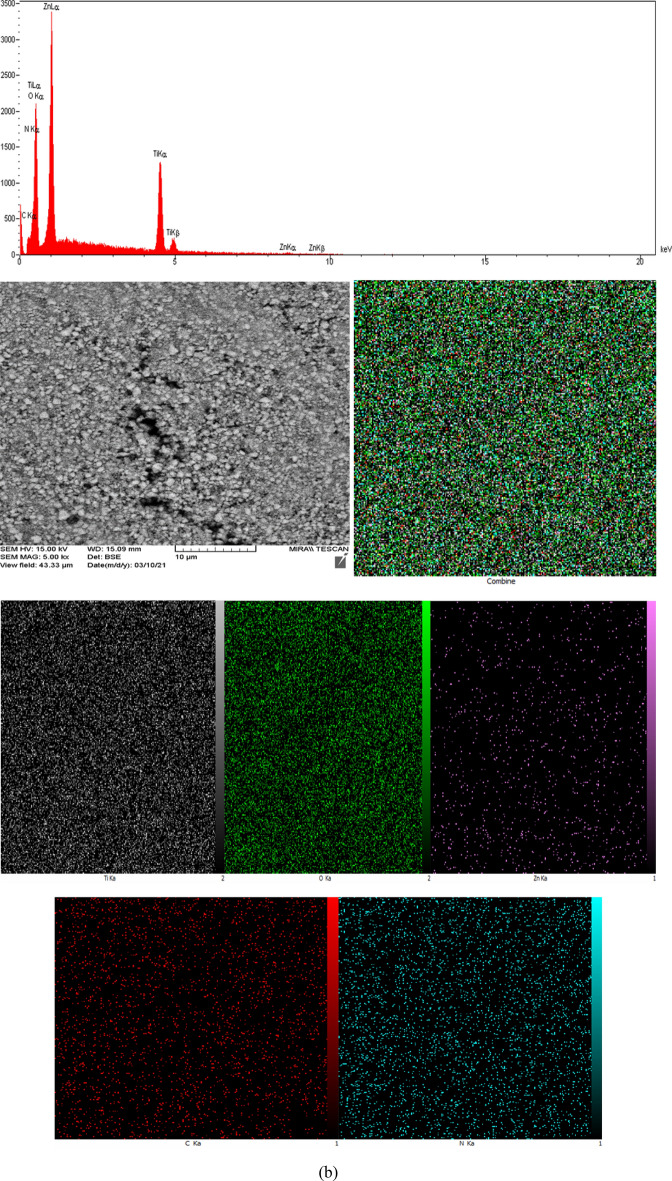

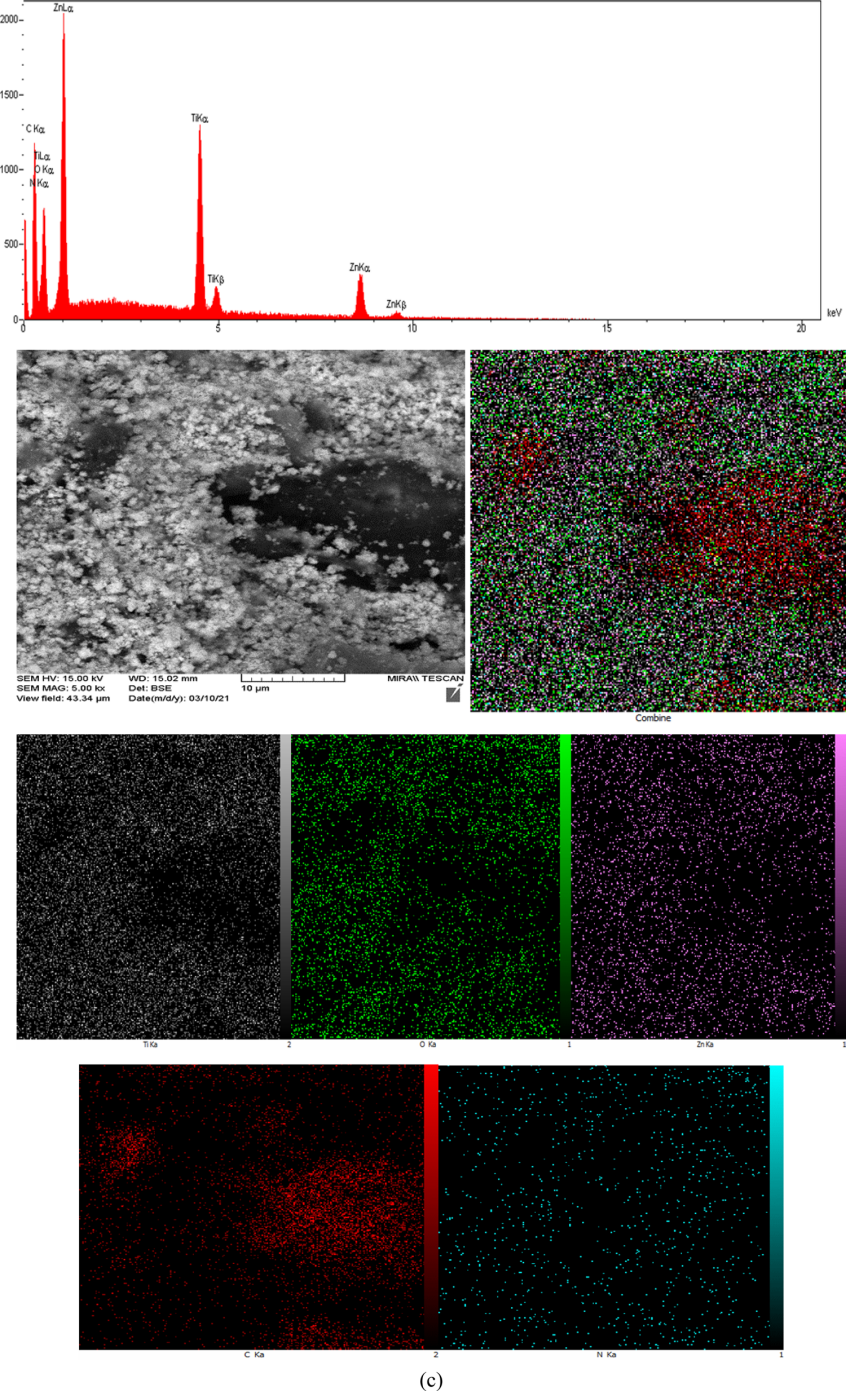
EDX spectra and corresponding mapping for (**a**) T1‒Z1/CS (**b**) T2‒Z1/CS (**c**) T1‒Z1/CS1‒Gr2.

The determining elemental composition and relative quantities through EDX spectra and element mapping for T1–Z1/CS and T2–Z1/CS ternary composites and T1–Z1/CS1–Gr2 composite have been presented in Fig. 4a, b, c and Tables 1, 2, 3, respectively. The spectrum peaks and elemental mappings indicate that the Ti, Zn, O, C, and N elements exist in all three cases. This observation is consistent with the previous results and confirms the absence of any impurities. It is noteworthy that the element of N can arise from the amine group of CS, and the relevant peak can be caused by the excitation of X-rays by this biopolymer^28^. According to the EDX spectrum peaks of T1–Z1/CS1–Gr2, the peak related to the C element has appeared intense compared to the similar peaks in the ternary composites. Meanwhile, as expected the relevant mapping confirms this observation and suggests that the Gr played its role correctly. In the EDX analysis of the T2–Z1/CS sample, according to Table 2, no weight percentage has been obtained for elements of C and N, but these elements have appeared in the relevant mapping. It may because of the lower amount of the sample when analyzing that the EDX analysis could not report these substances. However, elemental mapping has presented the presence and dispersion of all five elements.

**Table 1:** Percentage of elements in T1‒Z1/CS.

Element	Weight %
C	5.40
N	7.43
O	37.70
Ti	23.28
Zn	26.19
Total	100.00

**Table 2:** Percentage of elements in T2‒Z1/CS.

Element	Weight %
O	67.14
Ti	30.07
Zn	2.80
Total	100.00

**Table 3:** Percentage of elements in T1‒Z1/CS1‒Gr2.

Element	Weight %
C	32.76
N	9.50
O	22.36
Ti	14.10
Zn	21.28
Total	100.00

**Figure 5:**
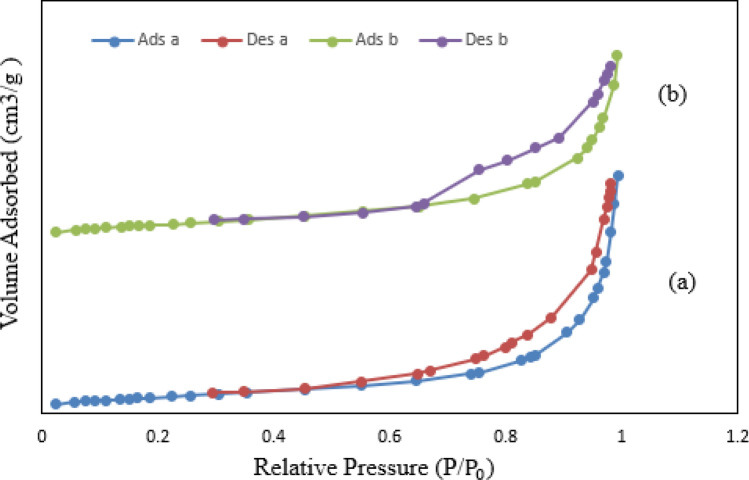
Nitrogen adsorption–desorption isotherms for (**a**) T1‒Z1/CS (**b**) T1‒Z1/CS1‒Gr2.

**Figure 6:**
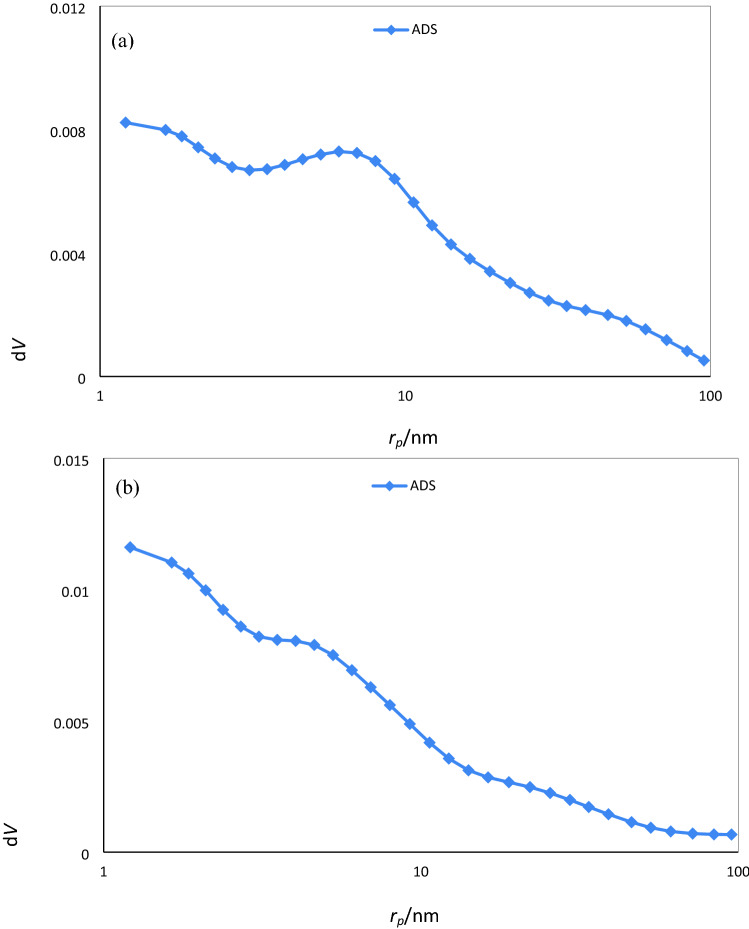
Pore size distribution for (**a**) T1–Z1/CS (**b**) T1–Z1/CS1–Gr2.

The studies of N_2_ adsorption–desorption isotherms (shown in Fig. 5) and pore size distribution (shown in Fig. 6) for T1–Z1/CS and T1–Z1/CS1–Gr2 composites exhibit a type IV isotherm and the mesoporous structure (2–50 nm) of samples, according to the IUPAC classification^19,41^. In addition, it is evident from the BET surface area results that the Gr presence provides a higher surface area, which envisaged that may lead to higher photocatalytic efficiency. In contrast, the ternary composite is superior regarding the mean pore diameter and total pore volume parameters according to the results of BJH analysis. It could be due to the pore-clogging occurrence by metal oxide particles in the T1–Z1/CS1–Gr2 sample, which they can present within the pore of Gr and at the surface of it simultaneously^42^; the other reason could be the inhibitory effect of CS on the porosity properties of composite^41^. The numerical results have been summarized in Table 4, revealing that the synthesized samples possess a desirable porous structure.

**Table 4:** Compared BET and BJH results.

Sample	BET surface area (m^2^/g)	Pore volume (cm^3^/g)	Mean pore size (nm)
T1‒Z1/CS	37.97	0.228	23.78
T1‒Z1/CS1‒Gr2	39.48	0.18	17.93

### Photodegradation

#### Photocatalytic activity

**Figure 7:**
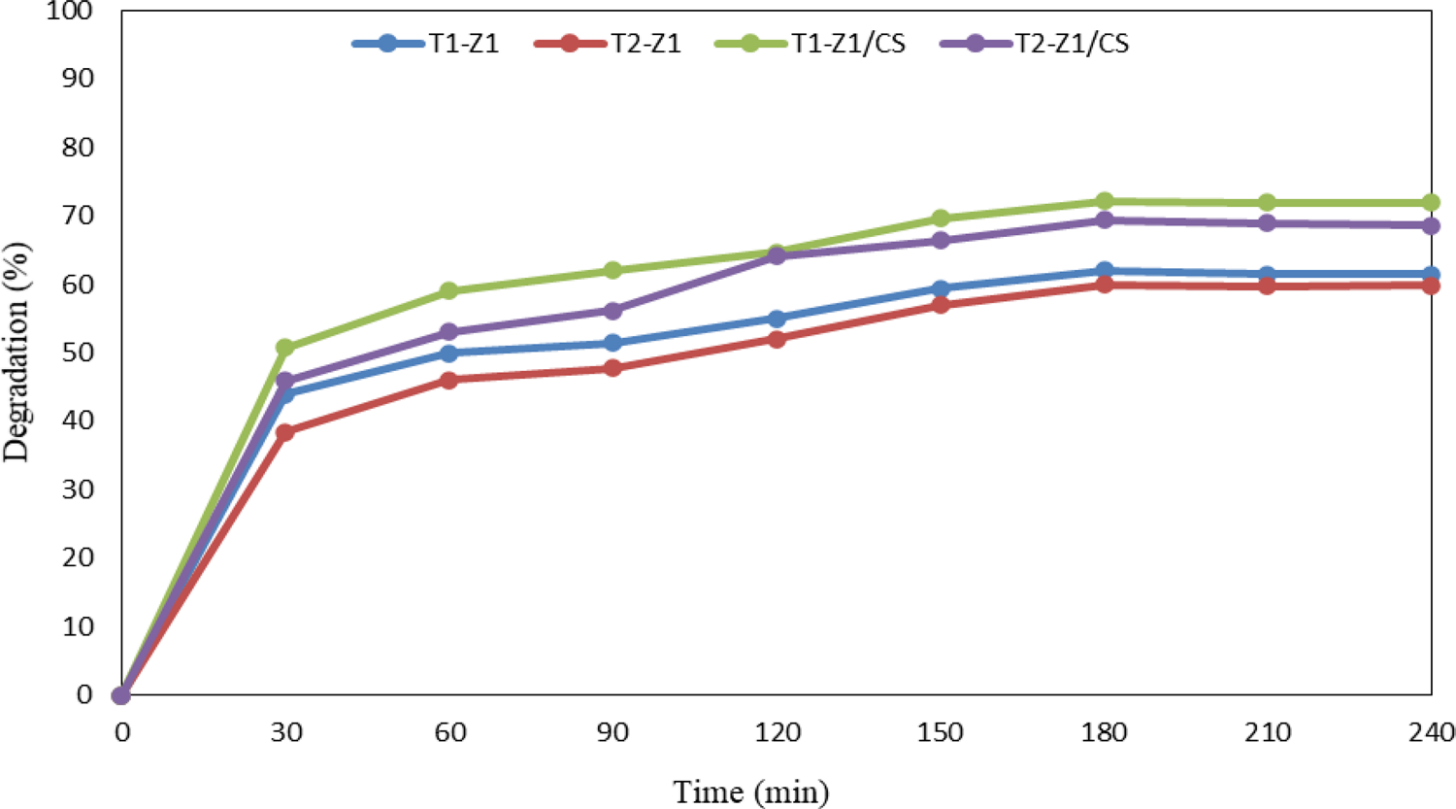
Photocatalytic activity of binary and ternary nanocomposites over time.

After assessing nanocomposites in terms of time-dependence photocatalytic activity, it was observed that in all four samples, the photocatalytic reaction reached equilibrium after 3 h, according to Fig. 7, and so no significant change in the degradation efficiency happened. This equilibrium time was considered as the default reaction time for other photocatalysts.

#### Determining most active photocatalyst

**Figure 8:**
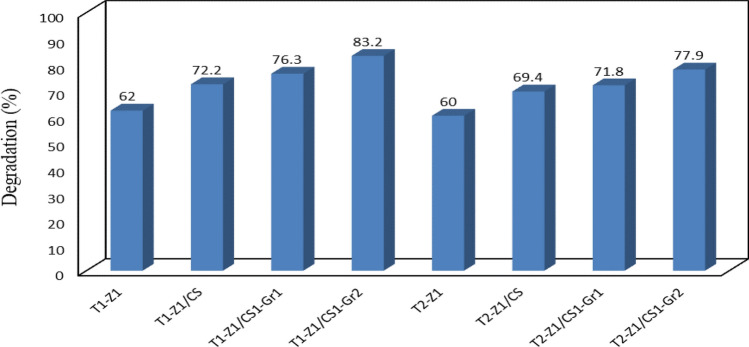
Comparison of photocatalytic activity of the all samples in a default condition.

The best composite was determined in terms of ratios between components in the constant default operational conditions. Results of evaluation showed that generally, the T‒Z nanocomposite exhibits the better photocatalytic performance with a 1:1 molar ratio compared to the 2:1 molar ratio. In addition, it was found that the higher amount of Gr in the composite leads to better acting in degrading of pollutant via providing a large specific surface area and possessing an excellent electron conductivity. That means Gr can accept or transfer the electrons in the photocatalytic process^15^. Figure 8 display these findings through a bar chart.

#### Effect of pH

**Figure 9:**
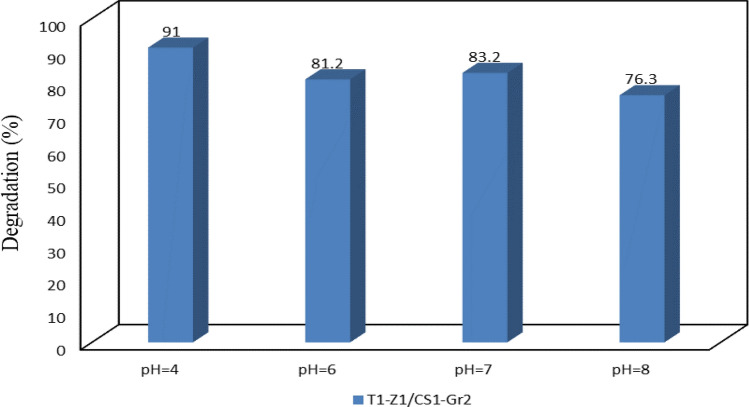
Photodegradation of TC via best nanocomposite at different pH values.

The pH parameter has a significant effect on the photocatalytic performance and surface properties of the relevant photocatalysts. Besides, the considered pollutant in this research (TC) is well known as an amphoteric molecule; in other words, TC can be cationic in pH values under 3.3 and anionic in pH values above 7.68. The desirable form of TC for enhanced sorption and photodegradation is the molecular form, available in the form of zwitterion TC in the pH range of 3.3–7.68 (acidic pH)^11,43^. It is noteworthy that the used metal oxide catalysts in this research have amphoteric behavior, too. They are positively charged in acidic conditions and conversely, according to the zero-point charges (pH_zpc_) for each case (about 6.5 for TiO_2_ and about 9 for ZnO)^6,11,44^. Here, it has been tried to select the pH values considering the properties of environmental water and TC pollutant reported by Liu et al.^43^. As observing in Fig. 9, the highest efficiency was obtained via the best nanocomposite at pH 4 in which both metal oxides have a positive charge on their surface. In addition, degradation efficiencies at pH values of 6 and 7 were obtained with a slight superiority at pH of 7; it can be caused by the change in surface properties of catalysts and TC. Finally, pH of 8 was selected for assessing the photocatalytic performance in the alkaline condition, where a considerable decrease in degradation efficiency has happened. It can result from negative surface charges of both TiO_2_ and TC, change in the number of hydroxyl radicals on metal oxides with the change in pH value, and control of reactants type and product type by the solution pH through the relevant ${\mathrm{pK}}_{\mathrm{a}}$ values^11^.

#### Effect of initial TC concentration

**Figure 10:**
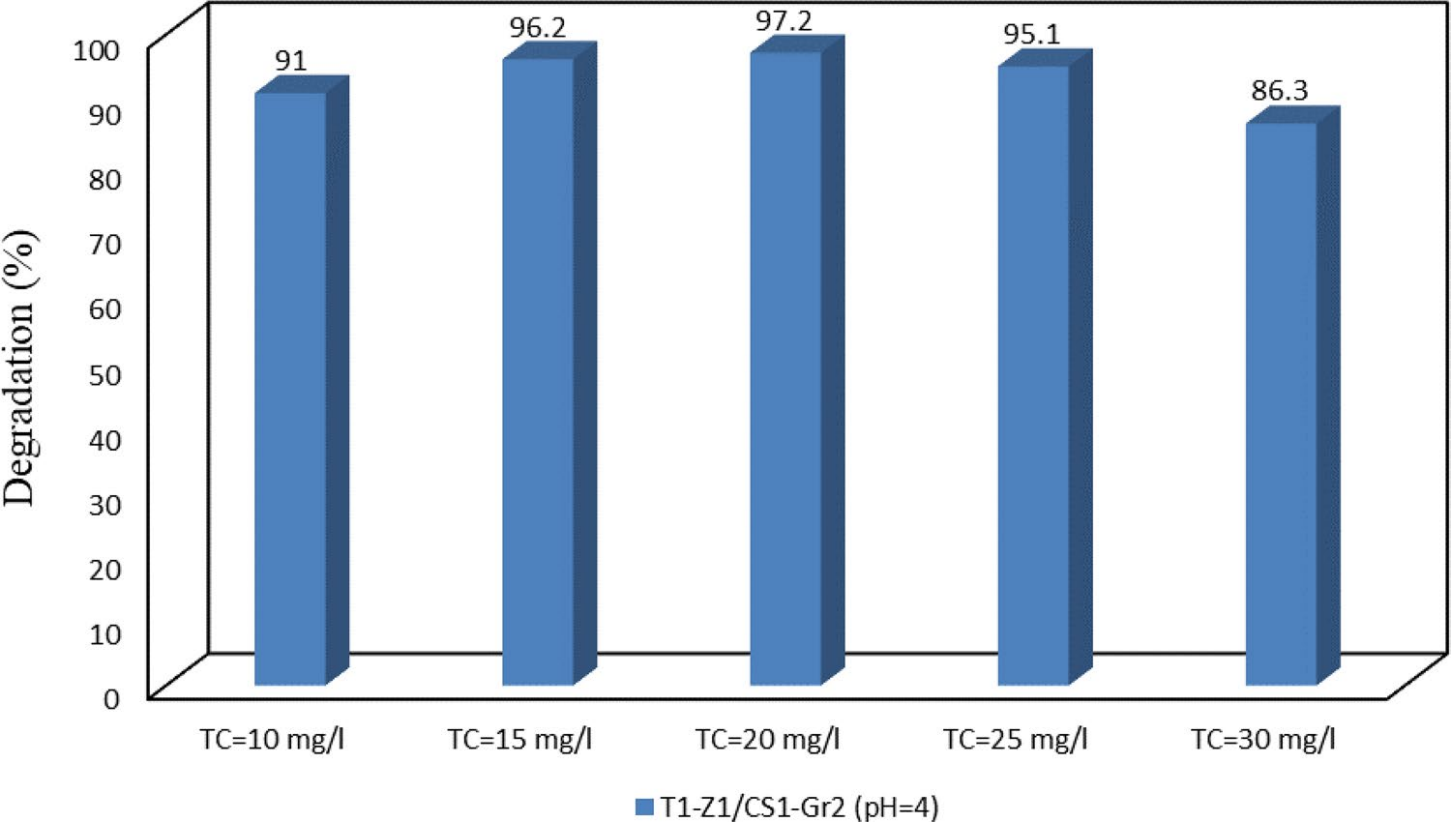
Photodegradation of TC with different concentrations at optimal pH.

After determining the optimum solution pH, four other TC concentrations were applied in addition to the default concentration (10 mg/L) to get the more appropriate TC concentration for degradation purposes. So, investigation results (Fig. 10) show that the increase in the initial TC concentration from 10 to 15 mg/L and 20 mg/L leads to improved degradation. However, with the increased concentration to above 20 mg/L, degradation efficiency took a declining trend where at the 30 mg/L concentration, more than a 10% decrease in efficiency was observed. This decrease can be due to the effect of TC and intermediate metabolites during the degradation process on the photocatalytic reaction through the adsorption of UV light^11^. Although at the 20 mg/L concentration the highest degradation was obtained, the main nanocomposite at the optimal pH showed acceptable efficacy for the removal of TC in the concentration range of 15–25 mg/L.

#### Effect of catalyst dosage

**Figure 11:**
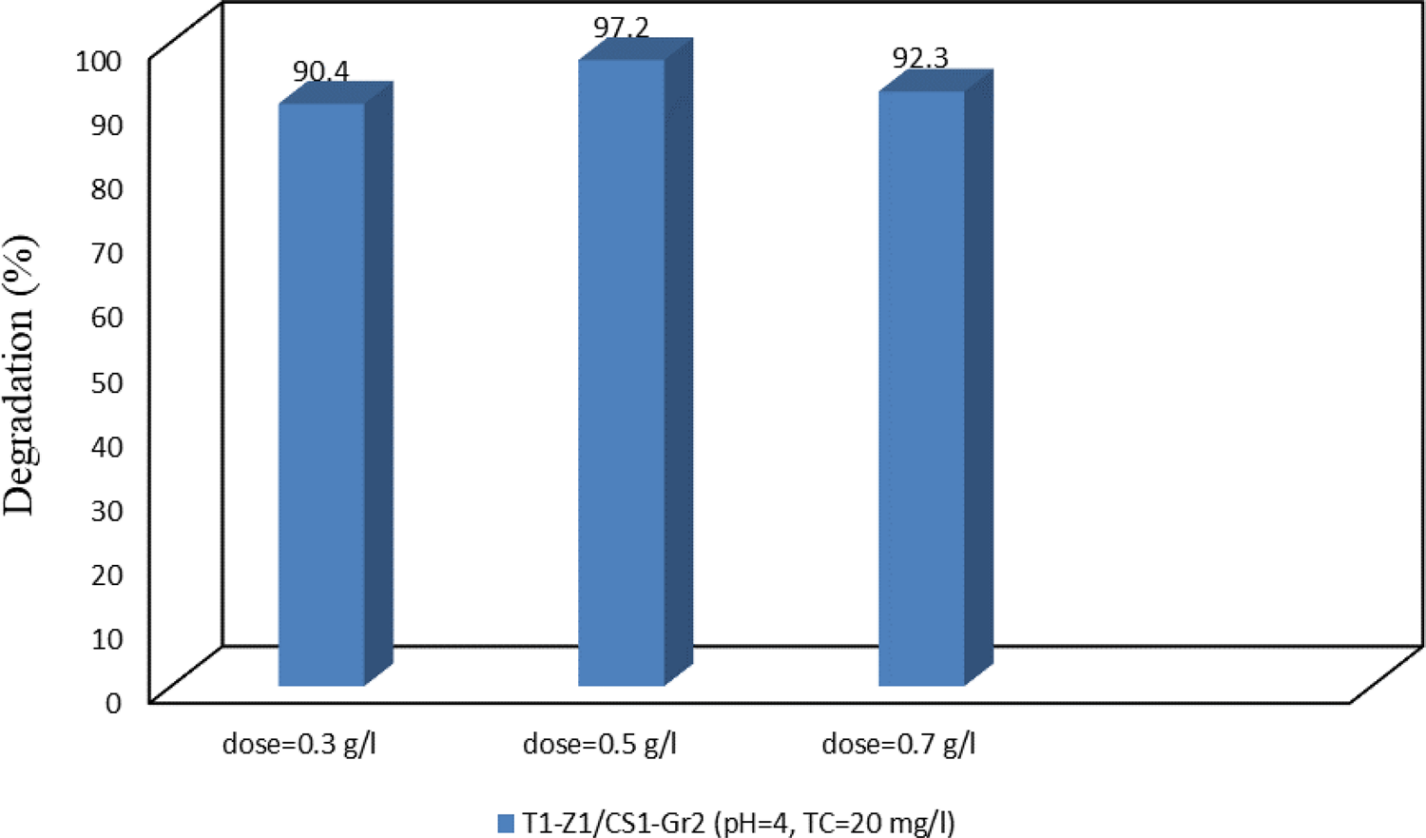
Photodegradation of TC at the optimized solution with different catalyst dosage.

After optimizing the parameters of the pollutant solution, variation in the dosage of the main photocatalyst was applied. Results show that the TC could be degraded in a lower percentage with the decrease for catalyst from default 0.5 g/L to 0.3 g/L (Fig. 11). A decrease in the number of potential photocatalytic sites on the surface of used photocatalyst can be the reason for the decline in efficiency^11^. In addition, an increased amount of catalyst to 0.7 g/L led to decreased efficiency. It can be due to the light scattering by the agglomeration of particles at the high dosage of catalyst. In other words, it might be interference that homogeneous structure of suspension, and reduction in the specific activity of the photocatalyst, and the number of active sites might occur in consequences^11^. Therefore, the default catalyst dosage of 0.5 g/L was determined as the optimum dosage in this study. The photocatalytic efficiencies of TC degradation of this study and other previously reported works have been compared in Table 5.

**Table 5:** Comparison of efficiencies for photocatalytic degradation of TC.

Photocatalyst	Light source	TC concentration (mg/l)	Photocatalyst dosage (g/l)	Reaction time (min)	Removal (%)	References
N, S-doped TiO_2_/CS	Visible-light	10	0.6	20	91	^7^
Calcite/TiO_2_	UV	50	1.5	300	90	^42^
ZnO/γ-Fe_2_O_3_	UV–visible light	30	0.5	150	88.5	^20^
C–N–S tridoped TiO_2_	Visible-light	5	0.5	180	97	^27^
TiO_2_ (P25)	UV	40	1	40	95	^45^
Carbon dots/high-crystalline g-C_3_N_4_	Visible-light	10	0.4	120	86	^46^
N-defected g-C_3_N_4_	Visible-light	10	0.5	120	97	^47^
Ag_3_PO_4_/Co_3_(PO_4_)_2_/g-C_3_N_4_	Visible-light	10	0.5	120	88	^48^
TiO_2_–ZnO/CS–Gr	UV	20	0.5	180	97.2	This Work

#### Photocatalytic degradation mechanism

The energy levels of the TiO_2_–ZnO catalyst as an influencing factor in photocatalytic processes, can be expressed that the electrons (e^−^) in the valence band (VB) of TiO_2_ are excited when irradiation of light with an appropriate wavelength with adsorbing of photon energy equivalent to the bandgap of TiO_2_. These electrons are promoted to the conduction band (CB) of ZnO due to the smaller bandgap of TiO_2_ against ZnO (3.20 eV for TiO_2_ and 3.37 eV for ZnO) and then leaving holes with positive charge in the VB of TiO_2_. The CS and Gr as solid supports provide a big light exposure area by providing a large surface and consequently better dispersal of used metal oxides. These improvements finally lead to more generation of electron–hole pairs and high degradation efficiency. Besides, it might be easily inhibited the recombination of generated electrons and holes due to CS and Gr presence because of the easy transfer of photogenerated electrons to the composite surface. The degradation is carried out by oxidizing the TC molecules that the ·O_2_^−^ and ·OH radical oxidative species doing this task. So, the photogenerated electrons react with O_2_ and generate ·O_2_^−^, while the holes generate ·OH radicals by reacting with OH^−^ or H_2_O at the surface^15,20^. It is noteworthy that because of hydroxyl groups of CS structure, it can degrade TC directly but not as much as TiO_2_ and ZnO^24,49^.

## Conclusions

The T1‒Z1/CS1‒Gr2 novel photocatalyst nanocomposite had the highest efficiency (97.2%) in the degradation of tetracycline under UV light after 3 h, amongst the multiple synthesized materials in this study. This superiority has resulted from the assigned 10 wt.% of the whole composite to the Gr, where its sheets improved the degradation activity by providing a higher surface area and better optical response properties, as well as solving the bandgap problems. Moreover, the results of characterization techniques and performed photodegradation tests complied together. Utilizing this optimized nanocomposite under visible light can be a promising approach in degrading TC or other various organic pollutants as purposes of another study. Besides, further reduction of agglomeration tendency of used metal oxides can be investigated by applying variation in the ratio of support materials.
